# Data for iTRAQ-based quantitative proteomics analysis of different biotypes in *Echinochloa crus-galli* with multi-herbicide treatment

**DOI:** 10.1016/j.dib.2016.10.017

**Published:** 2016-10-26

**Authors:** Xia Yang, Zichang Zhang, Tao Gu, Mingchao Dong, Qiong Peng, Lianyang Bai, Yongfeng Li

**Affiliations:** a Institute of Plant Protection, Jiangsu Academy of Agricultural Sciences, Nanjing 210014, China; b Biotechnology Research Center, Hunan Academy of Agricultural Sciences, Changsha 410125, China

## Abstract

Barnyardgrass (*Echinochloa crus-galli*) is one of the most troublesome herbicide-resistant weeds worldwide that interferes with rice growth and rice yield. Here we provide the data from a comparative proteomic analysis of leaves in resistant (R) and susceptible (S) biotypes of *Echinochloa crus-galli* both with and without multi-herbicide treatment in two independent biological experiments using iTRAQ. The distribution of length and number of peptides, mass and sequence coverage of proteins were presented, and the repeatability of the replicates was analyzed. 1342 differential accumulated proteins were identified from 2248 unique peptides by searching uniprot database and data analysis. These results are associated with the research article "Quantitative proteomics reveals ecological fitness cost of multi-herbicide resistant barnyardgrass (*Echinochloa crus-galli* L.)" (X. Yang, Z. Zhang, T. Gu, M. Dong, Q. Peng, L. Bai, Y Li, 2017) [1].

**Specifications Table**

**Table:** 

Subject area	*Biology*
More specific subject area	*Plant proteomics*
Type of data	*Excel files, figures*
How data was acquired	*iTRAQ, mass spectroscopy, instruments including* AKTA purifier 100 system, EASY- nLC 1000 HPLC system, Q-Exactive mass spectrometer
Data format	*Analyzed*
Experimental factors	*The barnyardgrass seedlings of herbicide-resistant and susceptible biotypes were treated with quinclorac, penoxsulam and bispyribac-sodium herbicides.*
Experimental features	*An equal amount of total proteins was prepared from the treated and untreated seedlings in the resistant and susceptible biotypes of Echinochloa crus-galli.*
Data source location	*Nanjing, China*
Data accessibility	*Data are available in this article.*

**Value of the data**

• The first large-scale proteomic data for barnyardgrass provide a fundamental basis for weed research.
• A total of 1342 proteins were identified from barnyardgrass seedlings of resistant and susceptible biotypes using iTRAQ.
• The data provide new insight into resistance mechanism and ecological fitness mechanism in herbicide-resistant barnyardgrass.

## 1 Data

**Fig. 1:**
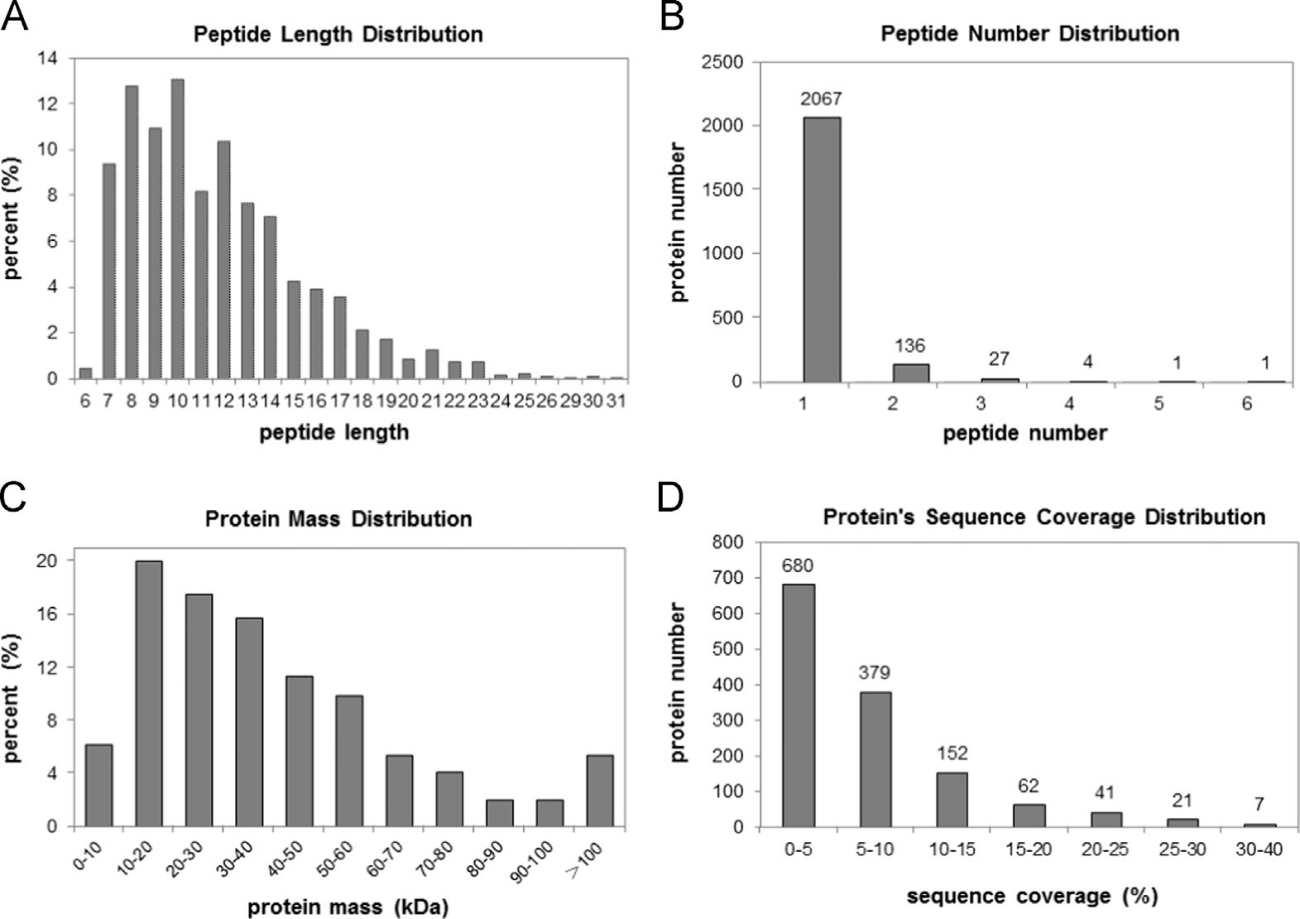
The distribution of length and number of peptides, mass and sequence coverage of proteins identified from iTRAQ proteomics.

**Fig. 2:**
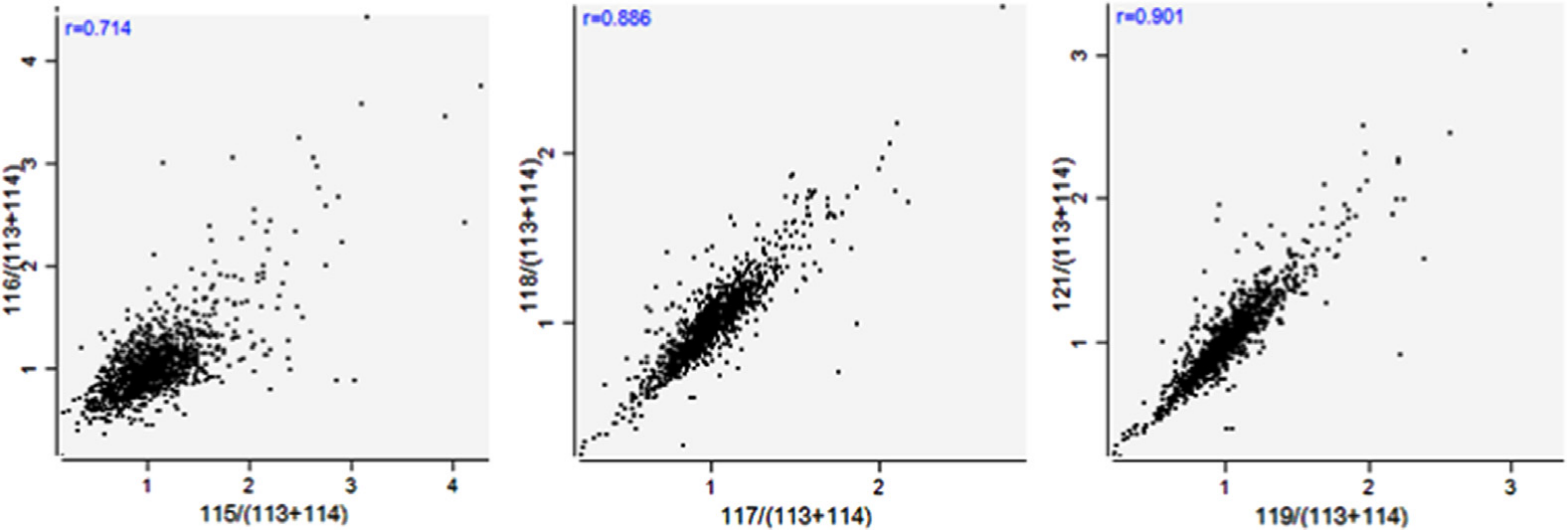
Scatter diagram of Pearson correlation between two replicates of iTRAQ test.

The distribution of length and number of peptides, mass and sequence coverage of proteins were presented (Fig. 1). A total of 1342 protein species (Supplementary Table 2) were identified from 2248 unique peptides (Supplementary Table 1). Prior to performing comparative analysis, Pearson׳s correlation of two biological replicates of iTRAQ test for each biotype/condition was performed to determine the analytical reproducibility (Fig. 2).

## 2 Experimental design, materials and methods

**Fig. 3:**
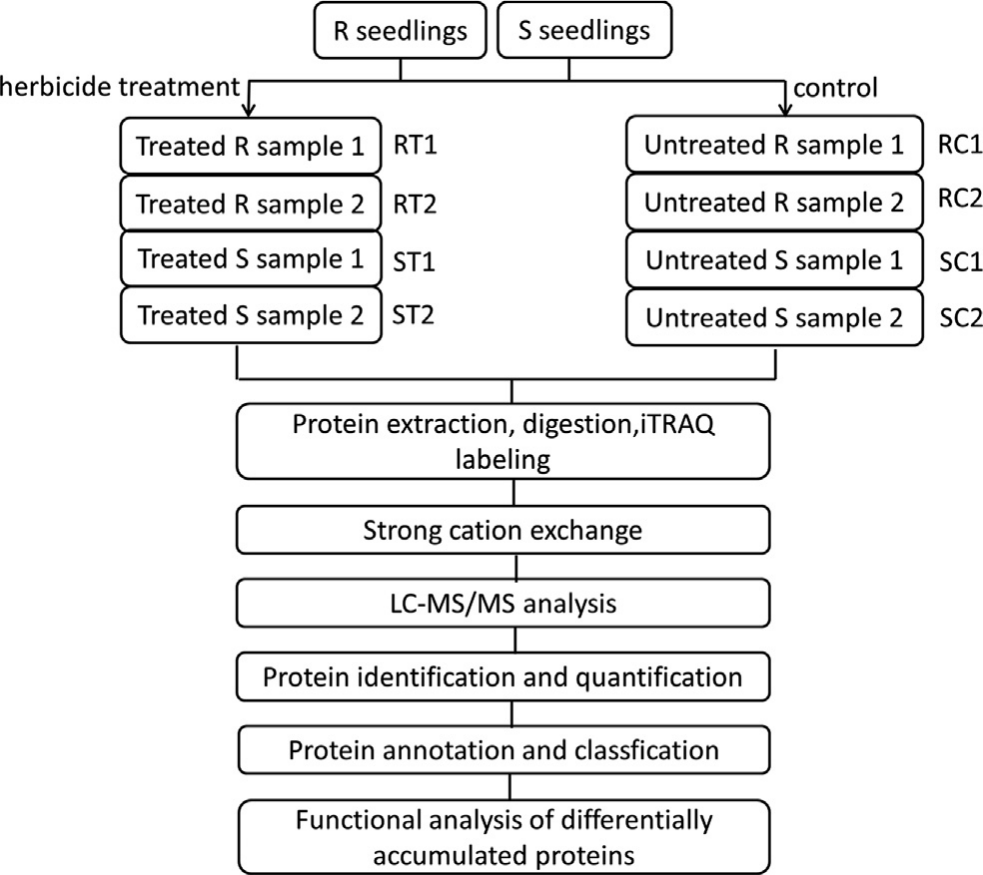
Flow chart of experimental design for the quantitative proteomics study.

Barnyardgrass (*E. crus-galli*) seeds from herbicide-susceptible (S) or -resistant (R) biotypes were processed as previously described [2]. For iTRAQ experiments and functional analysis, a flow chart related to the associated research article [1] was shown in Fig. 3.

### 2.1 Protein extraction

Total proteins were extracted from the herbicide-treated and untreated seedlings of the resistant and corresponding susceptible biotypes in *Echinochloa crus-galli*. Each sample was gound to fine powder with a pestle in liquid nitrogen, and precipitated with 25 mL buffer (TCA/acetone (1:9), 65 mM DTT) at −20 °C for 1 h, followed by centrifugation at 10,000 rpm for 45 min. The acetone precipitated sample was lysed in STD buffer (4% SDS, 150 mM Tris–HCl, 1 mM DTT, pH 8.0). The ratio of buffer to sample was 10:1 (v/v). After vortex mixing and a boiling water bath for 5 min, the suspension was ultrasonicated under the power of 80 w for 10 times (duration: 10 s, time interval:15 s) and then incubated in a boiling water for 5 min. The crude extract was clarified by centrifugation at 14,000*g* for 10 min. Thereafter, the supernatant was collected and protein content was measured by bicinchoninic acid (BCA) assay [3].

### 2.2 Protein digestion and iTRAQ labeling

150 μg protein of each sample was diluted with 200 μL UA buffer (8 M Urea, 150 mM Tris–HCl (pH 8.0)) and then loaded on a 10 KDa ultrafiltration filter (Sartorius, Germany). After 15 min centrifugation at 14,000*g*, another 200 μL UA buffer was added to the filter and centrifuged again at 14,000*g* for 15 min. Then, 100 μL iodoacetamide (50 mM in UA buffer) was added to each filter and mixed for 1 min at 600 rpm. The mix was incubated in darkness for 30 min and then centrifuged at 14,000*g* for 10 min. The filter was washed twice with 100 μl UA buffer and centrifuged for 10 min at the same condition. Subsequently, 100 μL dissolution buffer (AB SCIEX, USA) was added to the filter and centrifuged for 10 min at the same condition. This step was also repeated twice. Finally, 2 μg trypsin (Promega, USA) in 40 μL dissolution buffer was added to each filter, mixed for 1 min at 600 rpm and digested at 37 °C for 16–18 h. The filter unit was transferred to a new tube and centrifuged at 14,000*g* for 10 min. The supernatant was collected and the resulting peptide content was measured at 280 nm [4]. About 80 μg peptides of each trypsin-digested sample was labeled using the iTRAQ Reagent-8plex Multiplex Kit (AB SCIEX, USA) as previously described in the associated research article [1]. The labeling solution reactions were incubated at room temperature for 1 h.

### 2.3 Strong cation exchange chromatography separation

All procedures are described in the associated research article [1].

### 2.4 Nano LC-MS/MS analysis and data analysis

All procedures are described in the associated research article [1].
