# Supplementary material for: Data for iTRAQ-based quantitative proteomics analysis of different biotypes in Echinochloa crus-galli with multi-herbicide treatment
Source: Data Brief. 2016 Oct 26;9:741–5. doi: 10.1016/j.dib.2016.10.017 (PMC5094685; doi:10.1016/j.dib.2016.10.017)
Supplement: Supplementary file 1 — Supplementary material [file mmc1.doc]

## Conflict of interest

The authors declare no competing financial interest.
